# Psychosocial Disparities Among Racial/Ethnic Minority Transgender Young Adults and Young Men Who Have Sex with Men Living in Detroit

**DOI:** 10.1089/trgh.2016.0027

**Published:** 2016-12-01

**Authors:** José A. Bauermeister, Tamar Goldenberg, Daniel Connochie, Laura Jadwin-Cakmak, Rob Stephenson

**Affiliations:** ^1^ School of Nursing, University of Pennsylvania, Philadelphia, Pennsylvania.; ^2^ School of Public Health, University of Michigan, Ann Arbor, Michigan.

**Keywords:** gender, health equity, minority, resilience

## Abstract

**Purpose:** Transgender populations in the United States experience unique inequities in health and social well-being; however, they continue to be categorized with men who have sex with men (MSM) in HIV surveillance. To illustrate the differences in the lived realities of young MSM and transgender youth, we compare psychosocial outcomes across a sample of transgender and MSM youth from Detroit.

**Methods:** Data for this study come from a community-based cross-sectional survey of young adults (ages 18–29) living in Detroit who identify as transgender and/or as cisgender young men who have sex with men (YMSM). Using participants' geographic location within the city of Detroit, we matched transgender participants (*N*=26) to YMSM (*N*=123) living in the same area, and compared the prevalence in risk and resilience indicators across the two groups.

**Results:** Transgender participants were more likely than YMSM to experience socioeconomic vulnerability across several indicators, including lower educational attainment and workforce participation, greater residential instability, and higher lifetime experiences of transactional sex. Transgender participants were more likely than YMSM to report poorer health status, higher symptoms of depression and anxiety, and greater experiences of daily hassles and gender-related discrimination. Transgender participants did not differ from YMSM peers on health-promotive factors, including self-esteem, coping mastery, purpose in life, or social support.

**Conclusions:** Our findings underscore the importance of addressing the social and economic inequities experienced by transgender young adults. Local- and national-level programmatic and policy interventions are recommended to alleviate the psychosocial vulnerability experienced by transgender young adults and to improve their health and social well-being.

## Introduction

Transgender individuals in the United States experience a high prevalence of targeted stigma and discrimination.^1,2^ The experience of stigma and discrimination marginalizes transgender individuals, resulting in an increase in a range of psychosocial stressors (e.g., poverty and homelessness) as well as negative health outcomes (e.g., substance use and psychological distress).^2,3^ To create effective programming for transgender communities, it is essential to understand the specific needs and lived realities of these communities as an essential part of the U.S. public health discourse.^4^ However, transgender individuals are often misgendered in public health surveillance, resulting in a dearth of knowledge about the health of transgender populations.^5^

For example, within the HIV literature, transgender women are recognized to account for a disproportionate number of new HIV infections compared with other risk populations, yet they are often incorrectly coded as “men who have sex with men (MSM)” in surveillance data. Although transgender populations and MSM may share similar psychosocial vulnerabilities (e.g., stigma, discrimination, and social marginalization) on account of their sexual and/or gender minority status within a cisgender heteronormative society, the prevalence and cumulative exposure to these risk factors are likely different across the two groups. Similarly, the increasing availability of resources geared to sexual minorities may not necessarily be responsive to the needs of gender minorities.^1,3^

This article attempts to unpack the experiences of stigma and discrimination among transgender young adults and adult cisgender young MSM (YMSM). These two groups are commonly combined as one risk group; however, in this analysis we sought to compare the psychosocial stressors, resources, and health outcomes between cisgender YMSM and transgender young adults to document the differences and to emphasize the research and programmatic importance of treating these two groups uniquely.

Transphobic discrimination can occur in many forms. Discrimination may include interpersonal discrimination, which often takes the form of victimization, and can occur as sexual, physical, and verbal violence and harassment.^2,6–8^ Transphobic discrimination in the United States also occurs at an institutional level with transphobic policies that limit the ability of transgender individuals to access employment, education, housing, healthcare, and other public services.^2,3^

According to the 2008 National Transgender Discrimination Survey, a U.S. community-based sample of 6450 transgender people, 90% of respondents reported experiencing harassment, mistreatment, or discrimination at work, 78% reported experiencing harassment at school, 19% reported being refused a home or apartment, and 19% said they were refused medical care because of their transgender identity.^2^ This survey also found vast racial disparities in the experience of transphobic discrimination in all of these settings, with transgender people of color reporting much higher rates of discrimination.^2^ These experiences highlight the importance of identifying and addressing the structural vulnerabilities experienced by transgender people.

Structural vulnerabilities, manifesting as experiences of stigma and discrimination, can lead to a number of psychosocial life stressors (e.g., poverty, homelessness, transactional sex, and incarceration) among transgender communities, and especially transgender youth.^2,3,9^ Garofalo et al. found that transgender youth in Chicago and Los Angeles experience disproportionate amounts of incarceration, homelessness, exchanging of sex for resources, forced sexual activity, difficulty accessing healthcare, and difficulty finding employment.

Transphobic stigma and discrimination, especially among youth, can result in familial rejection or abuse, which may result in housing instability.^3,10^ Discrimination and rejection at school are associated with greater school absenteeism and drop out among transgender youth.^3,11^ Transgender communities (especially transgender communities of color) also disproportionately experience police discrimination, incarceration, and victimization while incarcerated.^2,12^

Although most research on transgender health has not had a large enough sample to test causal pathways between psychosocial stressors and poor health outcomes, much research has hypothesized that health inequities experienced by transgender individuals result from simultaneous exposure to multiple psychosocial stressors.^13^ This body of research suggests that experiences of stigma and discrimination, as well psychosocial life stressors such as poverty and homelessness (which are exacerbated by stigma), can result in an increased vulnerability to a number of co-occurring and mutually reinforcing poor health outcomes, such as mental health and sexual health outcomes.^14–17^

A systematic review examining HIV among transgender women found that, among four studies that used laboratory testing to confirm HIV status, 27.7% of the overall sample and 56.3% of black transgender women were living with HIV.^6^ In addition, a study by Reisner et al.^18^ found that among 298 young transgender women, 41.5% had received a psychiatric or substance dependence diagnosis and 20.1% had two or more comorbid diagnoses, which highlights the co-occurrence of negative health outcomes.^18^

Furthermore, according to the 2008 National Transgender Discrimination Survey, 41% of respondents reported attempting suicide at some point in their lives, with higher reporting among participants who also stated that they experienced discrimination.^2^ Additional research has found that experiences of transphobia increase experiences of depression, whereas experiences of social support and immersion within communities of lesbian, gay, bisexual, and transgender (LGBT) individuals may be protective and reduce psychological distress.^2,19–21^ Therefore, to fully understand how experiences of discrimination influence the health of transgender communities, it is important to understand the negative effects of trans-related stigma.

Resilience is even more important for transgender youth who are at a unique stage of adolescent development.^22,23^ Scarce data documenting the psychosocial assets (i.e., intrinsic characteristics within an individual; e.g., self-esteem, coping mastery, and purpose in life) and resources (i.e., external positive factors within the individual's social context; e.g., social support and mentors) in transgender populations exist. Consistent with the larger resilience literature,^24^ it is vital that researchers consider the availability of promotive factors across multiple social settings (e.g., family, peers, and neighborhoods) as they may reduce the negative effects caused by risk factors, and inform strengths-based intervention strategies.

Furthermore, the conscious inclusion of resilience indicators in research strengthens our ability to document that transgender people have positive attributes in their lives, including hopes and dreams for the future rather than solely focusing on risk and negative outcomes. Consequently, we included the prevalence of health-promotive factors in our examination of transgender adolescents and young adults living in Detroit Metropolitan Area (DMA).

Although most transgender health research has focused on identifying risk correlates explaining the disparities in mental health, sexual health, or substance use outcomes between transgender and cisgender individuals, fewer studies have focused on the general experiences of health and psychosocial stressors.^13^ Furthermore, few studies have compared the lived experience of transgender youth with their cisgender counterparts, and importantly, have not compared whether transgender youth experience outcomes different from cisgender sexual minority youth, with whom they are often categorized in many routine data collection systems.^17^

To better understand the experiences of transgender individuals, this study examines a variety of psychosocial risk and promotive factors, health outcomes, and examines the differences between transgender youth and cisgender YMSM in Detroit, MI. This new knowledge has the potential to shape both research and programmatic priorities aimed at increasing the health of transgender youth in the United States.

## Methods

Data for this article come from a cross-sectional observational HIV study examining the structural and psychosocial vulnerabilities experienced by YMSM and transgender young adults in the DMA. Participants were recruited online and in person. On the Internet, advertisements were posted on Black Gay Chat Live and Facebook. In-person recruitment occurred across gay bars, clubs, and community events frequented by the target population, as well as by staff from community partner agencies, clinics, and other agencies in the DMA working with YGBM (i.e., LGBT organizations, AIDS service organizations, and community and university health clinics). Advertisements displayed brief information about the survey, a mention of a $30 VISA e-gift card incentive upon completion, and the survey's website.

Participants who logged onto the survey's website could complete a screener. Participants who identified as male or transgender, regardless of sex assigned at birth, were eligible to participate. Furthermore, eligible participants had to be between the ages of 18 and 29 (inclusive), report currently residing in the DMA (as verified by zip code and IP address), and report ever having had sex with men.

### Procedures

We developed our web survey using best practices,^25^ including various iterations of pilot testing before data collection. Study data were protected with a 128-bit SSL encryption and kept within our university's firewalled server. Upon entering the study site, participants were asked to enter a valid and private e-mail address, which served as their username. This allowed participants to save their answers and, if unable to complete the questionnaire in one sitting, continue the questionnaire at a later time.

Upon completing an eligibility screener, eligible youth were presented with a detailed consent form that explained the purpose of the study and their rights as participants, and were asked to acknowledge that they read and understood each section of the consent form. We acquired a Certificate of Confidentiality to protect study data. The University of Michigan Institutional Review Board approved all study procedures.

Consented participants then answered a 45–60-min questionnaire that covered assessments of their sociodemographic characteristics, HIV status, individual-level characteristics (i.e., sexual and substance use behaviors), perceptions and experiences with community (e.g., social networks, neighborhood, stigma, and participation in minority communities), general mood over the past few months, and their hopes and dreams. Participants were compensated through e-mail upon completion of the questionnaire. For those questionnaires that were incomplete, participants were sent two reminder e-mails that encouraged them to complete the questionnaire: one e-mail was sent a week after they had started the questionnaire and another was sent a week before the questionnaire was scheduled to close.

Data were collected between May and September 2012. We used best practices to identify duplicates and falsified entries by manually examining participants' online presence, e-mail and IP addresses, operating system and browser information, irregular answer patterns, and time taken to complete survey.^26,27^ We concluded with an analytic sample of *N*=461 sexual and gender minority youth, of which 32 were eligible and consented but did not commence the survey (i.e., a study completion rate of 93.05%).

From this data-set, we created a subset that included transgender individuals (*N*=26) and YMSM (*N*=123) for our subsequent analyses. Given the limited sample size of transgender participants, we were unable to stratify them as transgender men (*N*=7) and transgender women (*N*=19) in subsequent analyses. To reduce endogeneity effects, we selected transgender participants who lived in the city of Detroit and matched them to YMSM peers living within the same residential zip codes. We included only racial/ethnic minority YMSM participants as no white transgender participants who met the eligibility criteria completed the survey.

Our data set (*N*=149) represented 15 zip codes unique to the city of Detroit. The range of participants by zip codes living in that geographic area spanned between 4 and 22 residents. Within each zip code, the range of YMSM residents varied between 3 and 18; the range for transgender residents by zip code varied between 1 and 5.

### Measures

#### Demographic characteristics

Participants were asked to report their age, race/ethnicity, sex assigned at birth (“What sex were you assigned at birth?”), and current gender (“What is your gender?” in which options included male, female, transgender male, transgender female). Participants also reported whether they lived alone and their relationship status. Participants' employment status, insurance coverage, income, housing stability, and relationship status were also ascertained. Participants noted their highest educational attainment (1=less than high school, 2=high school or GED, 3=technical/associate degree, 4=some college, 5=college or graduate work). We dichotomized education into whether or not participants had completed high school.

Participants were also asked to report whether they had health insurance (“Are you covered by any health insurance?”). We also dichotomized participants' income into above or below the federal poverty line. Residential instability was ascertained by whether or not (0=no, 1=yes) participants had spent at least one night in the past 30 days in a shelter, public place not intended for sleeping (e.g., bus station, car, and abandoned building), on the street or outside, in a temporary housing program, or in a welfare or voucher motel.

#### Transactional sex

Participants were also asked to report their lifetime engagement in transactional sex for socioeconomic means within a main/regular partnership.^28^ They also answered their engagement in transactional sex within a casual partnership on a four-point scale (0=false; 3=true). Engagement was measured using four items for both casual and regular relationships: “paying for things that I couldn't afford by myself,” “having a place to live,” “paying for groceries, utilities, or other bills,” and “providing for someone else who depends on me for financial support.” A mean composite sum score was created for each relationship type (Regular partner: Cronbach's α=0.89 for transgender sample, α=0.95 for YMSM sample; Casual partner: Cronbach's α=0.92 for transgender sample; Cronbach's α=0.95 for YMSM sample).

#### Health and well-being

We used three of the standard health-related quality-of-life (HRQOL) indicators^29^ from the Behavioral Risk Factors Surveillance System. The first indicator, self-rated health status, was measured using a five-point ordinal scale (1=poor; 5=excellent). The other two HRQOL measures ask participants to note the number of days in the prior 30 days when their physical or mental health, respectively, had not been good.

The physical health item read as follows: “Now thinking about your physical health, which includes physical illness and injury, how many days during the past 30 days was your physical health not good?” Similarly, the mental health item asked, “Now thinking about your mental health, which includes stress, depression, and problems with emotions, how many days during the past 30 days was your mental health not good?”

Participants were also asked to report their current HIV status (0=negative, 1=positive, 2=unsure/unknown) as well as whether they had ever been previously diagnosed with a sexually transmitted infection (STI) (gonorrhea, syphilis, chlamydia, etc.) by a healthcare provider (0=no, 1=yes).

#### Psychological distress

We measured psychological distress using two validated scales for depression and anxiety symptoms. To ascertain depressive symptoms, participants were asked to report on a 10-item short form of the Center for Epidemiologic Studies Depression scale.^30^ Items (e.g., “I felt that everything I did was an effort”) were scored on a four-point scale: 0=rarely or none of the time (<1 day); 3=most or all of the time (5–7 days). The total score was calculated by reverse scoring positively worded items (e.g., “I felt hopeful about the future”) and creating a sum score. High scores indicated high depressive symptoms in the past week (Cronbach's α=0.79 for transgender sample, α=0.79 for YMSM sample). A binary variable was created if the participant reported a score greater than or equal to 9, representing clinical symptomology for depression.

Six items from the Brief Symptom Inventory^31^ were used to assess anxiety symptoms. Items were offered on a five-point scale (0=never to 4=very often) and summed together for a composite anxiety score (Cronbach's α=0.89 for transgender sample, α=0.92 for YMSM sample). To assess the clinical cut point, we transformed participants' raw sum scores into T-scores, in which scores greater than 62 were noted as meeting the clinical anxiety cut point. Participants also reported on whether a doctor, psychologist, or mental health professional had ever told them they had any mental health condition (0=no, 1=yes).

#### Stress and coping

We used the Cohen's Perceived Stress Scale^32^ to estimate participants' perceived stress (i.e., daily hassles; five items) and ability to manage stress (i.e., coping mastery; six items). Items were answered on a five-point scale (1=never; 5=very often). We computed a mean daily hassles score (Cronbach's α=0.88 for transgender sample, α=0.92 for YMSM sample) and a coping mastery score (Cronbach's α=0.89 for transgender sample, α=0.96 for YMSM sample).

#### Self-esteem

Self-esteem was measured using the 10-item Rosenberg Self-Esteem Scale.^33^ Participants responded to items (e.g., “I feel I have a number of good qualities”) on a four-point scale (1=strongly disagree; 4=strongly agree). We created a mean composite self-esteem score, in which higher scores indicated higher self-esteem (Cronbach's α=0.85 for transgender sample, α=0.84 for YMSM sample).

#### Purpose in life

We used eight items from Ryff's Scale of Psychological Well-being^34^ to ascertain participants' purpose in life. Participants answered each item (e.g., “Some people wander aimlessly through life, but I am not one of them”; “I have a purpose in my life that says a lot about who I am”) using a four-point scale (1=strongly disagree; 4=strongly agree). We created a mean composite score, in which higher scores indicated greater purpose in life (Cronbach's α=0.57 for transgender sample, α=0.67 for YMSM sample).

#### Resilience

We used 14 items from Connor–Davidson's Scale of Psychological Well-being^35^ to ascertain participants' purpose in life. Participants answered each item (e.g., “I am able to adapt to change”; “I am not easily discouraged by failure”) using a five-point scale (1=never true; 5=true nearly all the time). We created a mean composite score, in which higher scores indicated greater endorsement to resilience statements (Cronbach's α=0.94 for transgender sample, α=0.97 for YMSM sample).

#### Future orientation

To measure future orientation, we generated a list of 13 hopes and dreams and asked participants to rate their importance on a four-point scale (1=not at all important; 4=very important). Because these different hopes and dreams may not be true or desired by all participants, we did not attempt to make a composite score; instead we compared the importance of each hope and dream individually (see Table 2 for the wording used for the 13 items).

#### Social support

Parental support was an abbreviated version of Procidano & Heller's scale^36^ measuring five statements (1) “My caregiver enjoys hearing about what I think,” (2) “I rely on my caregiver for emotional support,” (3) “My caregiver is good at helping me solve problems,” (4) “I have a deep sharing relationship with my caregiver,” and (5) “I rely on my caregiver for moral support.” For item, participants indicated their level of agreement on a scale from 0 (false) to 3 (true). We created a mean score, in which higher scores indicated greater emotional support (Cronbach's α=0.85 for transgender sample, α=0.95 for YMSM sample).

Support from friends was examined through five items using a four-point scale (1=not true, 4=very true). These five items were (1) “I rely on my friends for emotional support,” (2) “My friends come to me for emotional support,” (3) “My friends are good at helping me solve problems,” (4) “My friends understand me,” (5) “My friends give me the moral support I need.” Higher scores indicated greater emotional support (Cronbach's α=0.92 for transgender sample, α=0.90 for YMSM sample).

Finally, participants were asked, “Is there an adult other than a parent or the person who raised you that you go to for support and guidance (i.e., a mentor)?” We created a dichotomous variable indicating whether a participant had a mentor.

#### Neighborhood context

We included two measures of perceived neighborhood context. The first measure, neighborhood support, assesses the way people feel about their neighborhood. Sample items include: “I like living in my neighborhood,” “If I needed advice about something, I could go to someone in my neighborhood,” “I believe my neighbors would help me in an emergency,” and “People in this neighborhood are welcoming to LGBTQ people.” Each of the seven items was assessed using a Likert scale (1=strongly disagree to 4=strongly agree). We created a mean composite score from these seven items, in which higher scores indicated greater perceived support from people in their neighborhood (Cronbach's α=0.89 for transgender sample, α=0.91 for YMSM sample).

The second neighborhood perception measure asked respondents to rate how often 10 delinquent activities occurred in their neighborhood (1=never to 4=often). Sample items of neighborhood crime included “Gunfire or shooting in your neighborhood,” “Prostitutes or cars driving through looking for prostitutes in your neighborhood,” and “Gang activity in your neighborhood.” We computed a mean composite score, with higher scores indicating greater perceived crime in the neighborhood (Cronbach's α=0.95 for transgender sample, α=0.95 for YMSM sample).

#### Discrimination

We measured interpersonal discrimination in the prior year using nine items measured on a four-point scale (0=never, 3=often). Sample items included whether participant had been “treated with less respect than others,” “called names or insulted,” and “threatened or harassed.” We created a mean composite score, in which higher scores indicated greater discrimination frequency in the prior year.

We assessed employment-related items from Herek's discrimination scale^37^ to measure sexuality-related work discrimination. Participants were asked to report whether they had experienced three work discrimination events (being denied employment or fired from a job, being denied a promotion or salary increase, and receiving an unfair work evaluation) as a result of their sexuality in the past year.

### Data analytic strategy

After conducting descriptive statistics of our variables of interest and assessing the psychometric properties of the scales used in this analysis, we performed bivariate analyses to examine differences by YMSM and transgender participants across our continuous (e.g., mean scores) indicators using Welch's *t*-test. For ease of comparison across measures, we ran unadjusted logistic regressions to examine whether there were statistically significant differences between YMSM and transgender participants. YMSM participants served as the referent group in these analyses. Given the exploratory nature of this study, our statistical significance criterion was set at *p*<0.10.

## Results

### Sample description

Twenty-six participants self-identified as transgender or had a gender identity that did not align with their assigned sex at birth. Among these transgender participants, 19 identified as women (12.8%) and 7 identified as men (4.8%); the remainder of participants identifying as cisgender males (*N*=123; 82.6%). Participants had a mean age of 22.57 years (standard deviation [SD]=2.80; median [M]=22). There were no age differences between YMSM (M=22.65, SD=2.85) and transgender (M=22.19, SD=2.55) participants (*t*=0.76, *p*=0.45).

The majority of the sample identified as black or African American (*N*=122; 81.9%); the rest of the sample identified as Latino (*N*=17; 11.4%) or mixed race (*N*=10; 6.7%). Most transgender participants identified as African American or black (*N*=22; 84.6%), mixed race/other (*N*=3; 11.5%), or Latino (*N*=1; 3.8%). YMSM were predominantly African American or black (*N*=100; 81.3%), Latino (*N*=16; 13.0%), or mixed race/other (*N*=7; 5.7%). We observed no differences in race/ethnicity between YMSM and transgender participants (χ^2^=2.70, *p*=0.26).

Forty percent of the sample reported living alone; transgender participants (*N*=13; 50%) were as likely as YMSM (*N*=47; 38.2%) to live alone (χ^2^=1.24, *p*=0.27). Forty percent of the sample reported being in a relationship (transgender: *N*=9; 34.6%; YMSM: *N*=50; 40.7%). We observed no differences in relationship status between YMSM and transgender participants (χ^2^=0.33, *p*=0.57).

### Socioeconomic position

Transgender individuals (*N*=18; 69.2%) were less likely than YMSM (*N*=111; 90.2%) to have completed a high school education (Table 1). Compared with YMSM peers, transgender young adults were 2.36 times less likely to have completed high school (*p*=0.01). Five transgender participants reported being currently enrolled in school (*N*=5; 20%): three were full-time and two were part-time students. Transgender participants also had 3.13 lower odds (*p*=0.03) of being currently enrolled in school compared with their YMSM peers (*N*=54; 43.9%).

**Table 1.** **Socioeconomic Position Between Transgender and Young Men Who Have Sex with Men Participants**

				95% CI		
	YMSM (*N*=123)	Trans (*N*=26)	OR	Lower	Upper	*p*
High school completion, *N* (%)	111 (90.2)	18 (69.2)	0.42	0.09	0.68	0.01
Currently in school, *N* (%)	54 (43.9)	5 (20.0)	0.32	0.11	0.91	0.03
Below federal poverty, *N* (%)	74 (62.7)	19 (73.1)	1.61	0.63	4.15	0.32
Currently employed, *N* (%)	68 (55.3)	9 (34.6)	0.43	0.18	1.04	0.06
Has employment benefits, *N* (%)	35 (51.5)	3 (33.3)	0.47	0.11	2.04	0.47
Workplace discrimination, *N* (%)						
Denied job	15 (12.2)	3 (16.7)	0.94	0.25	3.51	0.93
Denied promotion	3 (2.4)	2 (7.7)	3.33	0.53	21.03	0.20
Unfair evaluation	6 (4.9)	4 (15.4)	3.55	0.92	13.60	0.07
Residential instability, M (SD)	0.28 (0.63)	0.62 (0.90)	1.74	1.03	2.93	0.04
Transactional sex, M (SD)						
Regular partner	0.76 (1.06)	1.18 (1.02)	1.43	0.98	2.08	0.07
Casual partner	0.56 (1.00)	1.23 (1.24)	1.67	1.16	2.39	0.005

CI, confidence interval; OR, odds ratio; YMSM, young men who have sex with men.

Sixty-four percent of the total sample reported living under the federal poverty line, with no statistical differences observed between transgender (*N*=19; 73.1%) and YMSM (*N*=74; 62.7%) participants (Table 1). We observed a marginal trend suggesting that transgender young adults (*N*=9; 34.6%) were less likely to be employed than YMSM (*N*=68; 55.3%) peers (odds ratio [OR]=0.43, *p*=0.06). Only three transgender participants reporting that their occupation provided benefits such as health insurance, paid sick days, retirement benefits, or paid vacation days. Transgender participants (*N*=4; 15.4%) were also more likely than their YMSM peers (*N*=6; 14.9%) to perceive that they had received an unfair work evaluation (OR=3.55, *p*=0.07).

To assess recent residential instability, participants were asked to select the number of places where they had spent at least one night in the past 30 days. Just over one-third of the transgender sample (*N*=10; 38.5%) had at least one transient night in the month prior compared with YMSM peers (*N*=27, 22.0%). Transgender participants (M=0.62, SD=0.90) were more likely to report several days of residential instability in the prior 30 days compared with YMSM peers (M=0.28, SD=0.63; OR=1.74, *p*=0.04). Transgender participants were also more likely to report engaging in transactional sex than their YMSM counterparts (OR=1.43, *p*=0.07 for regular partners; OR=1.67, *p*=0.005 for casual partners).

### Health status and indicators of well-being

Transgender participants rated their overall health status between “Good” and “Very Good,” slightly lower (OR=0.65, *p*<0.07) than their YMSM peers (Table 2). In terms of sexual health, the majority of the sample reported being HIV negative (*N*=122; 82.4%). Among participants living with HIV, we observed no differences between YMSM (*N*=13; 10.7%) and transgender (*N*=2; 7.7%) participants. Four transgender participants (15.4%) reported being diagnosed with one or more sexually transmitted infections (STIs) in their lifetime as compared with YMSM (*N*=22; 18.3%). There were no statistical differences between transgender and YMSM participants regarding their HIV (χ^2^=0.83; *p*=0.66) or STI (χ^2^=0.02; *p*=0.89) status.

**Table 2.** **Health and Well-Being Among Transgender Young Adults and YMSM Peers**

				95% CI		
	YMSM (*N*=123)	Trans (*N*=26)	OR	Lower	Upper	*p*
Self-rated health, M (SD)	3.02 (0.91)	2.65 (0.89)	0.65	0.41	1.03	0.07
Functioning (past 30 days), M (SD)						
Physical health	0.68 (1.93)	1.30 (3.51)	2.53	0.68	9.45	0.17
Mental health	1.87 (5.27)	3.88 (8.13)	1.77	0.67	4.45	0.23
Psychological distress						
Depressive symptoms, M (SD)	10.60 (5.80)	13.28 (6.00)	1.08	1.00	1.16	0.04
Meeting criteria, *N* (%)	54 (50.9)	18 (72.0)	2.48	0.96	6.42	0.06
Anxiety symptoms, M (SD)	4.56 (5.55)	8.16 (5.49)	1.10	1.03	1.19	0.01
Meeting criteria, *N* (%)	11 (9.5)	3 (12.0)	1.30	0.34	5.06	0.70
Stress and coping, M (SD)						
Mastery	2.95 (1.10)	3.01 (0.81)	1.06	0.70	1.59	0.79
Hassles	2.42 (1.03)	2.87 (0.94)	1.55	1.01	2.37	0.04
Psychological well-being, M (SD)						
Self-esteem	3.00 (0.56)	3.10 (0.52)	1.37	0.63	2.98	0.43
Purpose in life	2.67 (0.78)	2.68 (0.59)	1.06	0.43	2.66	0.90
Resilience	3.76 (0.90)	3.71 (0.73)	0.93	0.57	1.52	0.77
Future orientation (hopes and dreams), M (SD)						
Continue schooling	3.59 (0.83)	3.48 (0.77)	2.10	0.85	5.19	0.11
Have your own business	3.31 (0.94)	3.28 (0.94)	1.20	0.50	2.86	0.68
Become a leader in your community	3.17 (1.00)	3.28 (0.89)	0.97	0.41	2.31	0.95
Be in a committed romantic relationship	3.32 (0.97)	3.40 (0.96)	0.83	0.34	2.04	0.69
Be politically involved in Detroit	2.72 (1.18)	3.08 (0.95)	0.83	0.34	2.02	0.68
Have children	2.90 (1.19)	3.24 (1.05)	0.62	0.26	1.47	0.28
Move to another safe	3.23 (1.04)	3.32 (0.90)	1.20	0.50	2.86	0.68
Feel safe	3.67 (0.76)	3.80 (0.50)	0.72	0.23	2.30	0.58
Own a home	3.62 (0.84)	3.44 (1.00)	1.44	0.54	3.85	0.47
Advocate for LGBT rights	2.89 (1.13)	3.32 (0.99)	0.47	0.20	1.14	0.09
Fight against racial discrimination	3.27 (1.00)	3.48 (0.77)	0.73	0.30	1.79	0.49
Be financially secure	3.68 (0.82)	3.80 (0.41)	1.18	0.39	3.50	0.77

Transgender participants and YMSM reported comparable physical and mental health in the prior 30 days (Table 2). Transgender participants (*N*=9; 32.1%) were more likely to report receiving psychological or emotional counseling in the prior year than YMSM (*N*=19; 15.6%), χ^2^=5.07; *p*=0.02.

Compared with YMSM peers, transgender young adults reported greater depression (M=2.24, SD=0.59 vs. M=1.96, SD=0.59; OR=2.11, *p*=0.04) and anxiety (M=2.34, SD=0.90 vs. M=1.80, SD=0.94; OR=1.72, *p*=0.01) symptoms (Table 2). Close to three-quarters of the transgender sample (*N*=18; 72.0%) reported symptoms above the clinical threshold for depression, being 2.48 times more likely than YMSM (*N*=54; 50.9%) peers (*p*=0.06) to have met clinical criteria for depression. Transgender participants (*N*=3; 12.0%) and YMSM (*N*=11; 9.5%) reported comparable symptoms meeting the clinical threshold for anxiety.

Transgender participants (M=2.87, SD=0.94) were more likely than YMSM (M=2.42, SD=1.03) peers to report a greater number of daily hassles (OR=1.55, *p*=0.04); however, these two groups did not differ in their mastery to cope with stress scores (Table 2). Furthermore, transgender young adults had scores comparable to YMSM in the self-esteem, purpose in life, and resilience scales.

Participants' perceived emotional support from their primary caregiver and from their peers was comparable between transgender and YMSM participants (Table 3). We also asked participants about mentors. Transgender young adults (*N*=5; 20%) and YMSM (*N*=20; 18.3%) reported having an adult other than a parent or the person who raised them that they go to for support and guidance.

**Table 3.** **Contextual Risk and Resilience Indicators Among Transgender Young Adults and YMSM Counterparts in Detroit**

				95% CI		
	YMSM (*N*=123)	Trans (*N*=26)	OR	Lower	Upper	*p*
Neighborhood context, M (SD)						
Neighborhood efficacy	2.23 (0.76)	2.17 (0.83)	0.90	0.51	1.59	0.72
Crime in neighborhood	2.72 (0.89)	3.10 (0.84)	1.72	0.99	3.00	0.05
Discrimination						
Frequency of discrimination, M (SD)	7.98 (7.25)	8.71 (8.42)	1.01	0.96	1.08	0.66
Gender identity discrimination, *N* (%)	22 (18.2)	17 (65.4)	8.50	3.35	21.56	0.001
Social support						
Family support, M (SD)	2.39 (0.80)	2.18 (0.76)	.074	0.40	1.36	0.34
Peer support, M (SD)	2.87 (0.76)	2.91 (0.78)	1.07	0.61	1.90	0.81
Availability of mentor, *N* (%)	28 (23.9)	5 (20.0)	0.80	0.27	2.31	0.67

### Interpersonal interactions

When asked to evaluate their neighborhood, transgender participants (M=2.17, SD=0.83) had scores comparable to YMSM peers (M=2.23, SD=0.76) regarding their perceived neighborhood support (Table 3); however, transgender youth (M=3.10, SD=0.84) were more likely than YMSM peers (M=2.72, SD=0.89) to report crime in their neighborhood (OR=1.72, *p*=0.05).

Experiences of discrimination in the prior year (e.g., being treated with less courtesy than others, being called names or insulted, and being threatened or harassed) were prevalent in the sample (Table 3). Experiences of gender-based discrimination were prevalent, with more than 65% of transgender youth reporting feeling discriminated because of their gender identity in the prior year. Transgender participants (*N*=17; 65.4%) were 8.5 times more likely than YMSM (*N*=22; 18.2%) to experience gender-based discrimination (*p*<0.001).

To learn about participants' hopes and dreams for the future, we finished the survey by asking them to indicate how important it would be for them to achieve a variety of future life goals (Table 2). Overall, transgender participants shared similar hopes and dreams for the future as YMSM; however, they were more likely to desire to advocate for LGBT rights (M=3.32, SD=0.90) than their YMSM peers (M=2.89, SD=1.13).

## Discussion

Incomplete knowledge about the health of transgender and other gender minority youth relative to their YMSM counterparts impedes our ability to understand and respond to their health and well-being needs, and limits our ability to advocate for key health priority areas in the U.S. public health agenda.^5^ In this study, we examined the prevalence of risk and resilience characteristics among transgender participants living in Detroit and subsequently compared their scores to their YMSM counterparts scores.

Consistent with prior research,^11^ transgender participants were less likely than their YMSM peers to have completed high school or currently be enrolled in school. These educational disparities are noteworthy given the substantial role that educational attainment in adolescence and early adulthood plays in the long-term social and financial advancement of populations. Compared with their YMSM peers, the proportion of transgender participants who reported working and receiving work-related benefits was much lower. They were also more likely to report an unfair work evaluation and, although not significant, they reported a greater prevalence of having been turned down for a job or a promotion.

These discrepancies in transgender young adults' experiences in the workforce might foreshadow the larger work-related inequities observed among older transgender populations.^2^ These socioeconomic inequities are particularly concerning, as nearly three-quarters of both transgender and YMSM participants reported living below the federal poverty line. Within this socioeconomic context, we noted that transgender participants reported greater residential instability in the prior 30 days and were more likely to have engaged in transactional sex with both casual and regular partners.

Taken together, these findings are concerning given the robust social epidemiologic data that illustrate that living in socioeconomically strained settings increases the propensity of earlier morbidity and mortality.^38,39^ Our findings underscore the importance of addressing the structural conditions that obstruct transgender young adults' socioeconomic position and social advancement and, consistent with prior literature, highlight the importance of developing strategies to promote the social and educational advancement of transgender populations as a public health approach.

Among health outcomes, transgender participants were more likely to report having poorer health than their YMSM peers. Transgender participants also reported greater symptoms of depression and anxiety, respectively, and greater daily hassles than their YMSM peers. Although transgender participants reported higher distress, we found no evidence to suggest that they had a greater probability of meeting depression or anxiety clinical criteria based on clinical cutoff points. In addition, we observed greater variability around the mean for transgender participants even if the group differences were not statistically significant (e.g., greater variability in the number of days in the prior month when transgender participants felt that their physical and mental health were not optimal).

It should be noted, however, that these indicators were not developed for transgender individuals and, although they had good psychometric properties in this sample, they may require validation and adaptation to fully capture the lived realities of transgender individuals. Alternatively, the discrepancy between symptomatology and meeting clinical criteria may be indicative of compensatory processes (i.e., active, chronic high-effort coping) on a day-to-day basis and, akin to high-effort active coping (e.g., John Henryism) among African American populations,^40^ may help explain the health disparities observed over time. At present, however, these ideas are exploratory and deserve greater attention in future research.

Transgender and YMSM young adults had comparable scores on various measures of intrinsic assets (e.g., self-esteem, coping mastery, resilience, purpose in life, and future orientation). Although these indicators are often linked to well-being in samples of adolescents and young adults, constraints in our sample size limit our ability to examine the role of these health-promotive assets on the health and well-being of transgender young adults through multivariable analyses, and highlight the need to recruit large samples of transgender individuals to test how these mechanisms may buffer the psychosocial vulnerability of transgender young adults in future research.

Furthermore, our limited sample size hindered our ability to examine differences between transgender men and transgender women. Future research examining disparities in psychosocial risks and resources within transgender populations is warranted.

Although both groups reported experiences of discrimination, transgender young adults reported a propensity to experience gender-related discrimination. Similarly, although both groups reported comparable scores regarding their neighborhoods' collective efficacy, transgender participants perceived their neighborhoods as more unsafe than their YMSM peers. Considering that we matched participants by zip code, these findings suggest that transgender young adults may perceive greater tension in their social environments, potentially because of the unique experiences of discrimination that they face because of their gender identity.

Nonetheless, we cautiously remind the reader that we are comparing two socially vulnerable groups to one another (i.e., YMSM are more likely to experience discrimination than their heterosexually identified counterparts in the literature^22^). Thus, the interpersonal tension observed among transgender and YMSM young adults in our sample is still potentially greater than that observed among cisgender heterosexual young adults in the same neighborhoods. Future research, both qualitative and quantitative, is necessary to understand how multiple marginalized identities create cumulative risks for diverse populations in the same geographic region.

Our study has several limitations. The limited sample size and cross-sectional design did not allow us to test more complex relationships between the indicators examined in this study. Future research with a larger sample of transgender youth, ideally in a longitudinal study, is warranted to examine how risk and resilience operate over time. Furthermore, contrary to other studies, transgender participants in our sample were not more likely to identify as living with HIV than their YMSM counterparts. It is possible that this difference is attributed to our community partners—as they provide HIV/STI testing in the metro region to these populations. Thus, self-selection bias to participate in the study could be present and may limit the generalizability of our findings.

Finally, our data focus on young adults between the ages of 18 and 29; it remains unclear whether similar findings would be observed in younger and/or older individuals. These limitations notwithstanding, our study had several strengths. First, our sample consisted of a large racially and ethnically diverse sample of transgender young adults who were matched to cisgender YMSM living in the same zip codes in the city of Detroit. In creating our matched comparison, we were able to reduce potential endogeneity biases. In addition, this study not only examined the overall prevalence of risk correlates, but also the health-promotive factors in their lives. Future research examining the concurrent presence of risk and resilience is warranted.

Although a number of studies have detailed the stigma and social vulnerability experienced by transgender individuals, the current article is unique in both its focus on young adults and its comparison with YMSM. The results illustrate the distinct social and economic disadvantage experienced by transgender young adults, and the associated increased experience in poor mental and physical health. Most apparent is the high level of stigma reported by transgender young adults, and it seems plausible to argue that this is a significant driving force behind much of the social and economic disadvantage they experience.

Although transgender young adults and YMSM share some of the same risks and vulnerabilities, the results highlight the need for research and programs that recognize and intervene on the unique structural inequities experienced by transgender young adults as a pathway to improving their socioeconomic, physical, and mental health.

## Abbreviations Used

GED: General Educational Development
HRQOL: health-related quality-of-life
MSM: men who have sex with men
OR: odds ratio
SD: standard deviation
STI: sexually transmitted infections
YMSM: young men who have sex with men
